# Effects of Lightning

**Published:** 1846-02

**Authors:** 


*Effects of Lightning.*—Our readers will doubtless recollect a remarkable paper, published in a former number of our Journal, entitled "extraordinary effects of a stroke of lightning," by John LeConte, M. D., of Savannah, Georgia. In a recent letter from the distinguished writer, we have the following additional particulars, in relation to some of the cases there mentioned:—

"Savannah, Dec. 4th, 1845.

"Dr. Lee: My Dear Sir,—Thinking that you might be interested in hearing something further concerning the history of the cases which formed the subject of my paper on the 'effects of lightning,' which was honored with a place in the 'New York Journal of Medicine, and the Collateral Sciences,' for Nov., 1844; I have concluded to avail myself of the privileges of a correspondent.

"You will recollect that only two out of the five negroes who were struck down, survived the shock, viz. cases four and five. Charlotte (case 4), up to the time she was struck by the lightning, had never been pregnant, although she was then past 29 years of age. The electricity seems to have produced a remarkable impression upon her uterine functions, causing a manifest derangement of the catamenial flux, as already noticed in my previous details of the case. During the early part of the last summer, she became pregnant for the first time in her life, and will shortly be confined!! She has been married about ten years. It is impossible to determine whether the establishment of the reproductive functions in this case, is in any manner connected with the powerful shock sustained by her nervous system; but, perhaps, the fact is not unworthy of note, when taken in connection with the extraordinary impression which was made upon the uterine functions in both of the survivors.

"Sarah (case 5), the woman past 70 years of age, in whom menstruation was re-established after twenty years' cessasion, still continues to menstruate with perfect regularity up to the present time, more than two years and five months since she was struck by lightning!! Her health still continues very good.—*N. Y. Jour. of Med.*

